# What Does a Swiss Franc Mortgage Cost? The Tale of Polish Trust for Foreign Currency Denominated Mortgages: Implications for Well-Being and Health

**DOI:** 10.1007/s11205-016-1363-9

**Published:** 2016-05-20

**Authors:** Piotr Białowolski, Dorota Węziak-Białowolska

**Affiliations:** 1 Department of Economic, Social, Mathematical and Statistical Sciences, University of Turin, Corso Unione Sovietica 218bis, 10134 Turin, Italy; 2 Econometrics and Applied Statistics Unit, European Commission Joint Research Centre, Via E. Fermi 2749, 21027 Ispra, Italy

**Keywords:** Causal effect, Carry trade, Panel data, Health, Mortgage debt, Well-being

## Abstract

It is commonly agreed that excessive household financial debts are detrimental to psychological and physical health. Research also demonstrates that housing instability, mortgage indebtedness and mortgage foreclosure negatively influence subjective well-being. In Poland at the beginning of 2015, homeowners with Swiss franc denominated mortgages suffered from an abrupt swing in the Swiss franc/Polish zloty (CHF/PLN) exchange rate, which resulted in considerable increase in the local currency value of their mortgages. These adverse financial circumstances were hypothesised to affect not only household finance but also negatively affect the psychological well-being and physical health of peoples. The 2013 and 2015 waves of the Polish representative household panel ‘Social Diagnosis’ were used to examine impact of the abrupt change in the CHF/PLN exchange rate in Jan. 2015 on well-being and health. Causal inference was investigated using a difference-in-differences matching estimator. Results showed that although impact of Swiss franc appreciation on the mortgage related financial burden was considerable, it did not affect well-being or health outcomes. Any manifestation of adverse effects was absent in the short term, which does not however preclude their long term existence.

## Introduction

There is a common agreement that debtors can experience significant risk and distress (Brown et al. 2005; Sweet et al. 2013). In particular, household financial debt is associated with adverse psychological (Bridges and Disney 2010; Selenko and Batinic 2011; Sweet et al. 2013) and physical health (Nettleton and Burrows 1998; Sweet et al. 2013). Research has also demonstrated that housing instability, mortgage indebtedness and mortgage foreclosure can be to the detriment of subjective well-being (Nettleton and Burrows 1998) and self-rated health (Cannuscio et al. 2012; Lau and Leung 2014). They may also result in increased propensity to anxiety attacks and depression (Burgard et al. 2012). Although these findings depict debts (mortgage included) in a negative light, indebtedness is important in fostering aggregated life-cycle utility (Friedman 1977; Modigliani and Brumberg 1954). Various studies have demonstrated that lack of access to credit, understood as financial exclusion, has a severe negative impact on household utility (Attanasio 1994; Crook 2003; Jappelli and Pagano 1989). It has also been found that households with mortgages, due to their relatively good financial situation, are unlikely to be excessively burdened with debt and frequently have a sufficient reserve to service their debts even in the face of adverse economic conditions (Białowolski 2014).

Relatively little is known, however, about the consequences of having mortgages denominated in foreign currencies on households. Such households, also referred to as carry traders^1^ (Beer et al. 2010; Galati et al. 2007), are numerous in almost all Central and Eastern European (CEE) countries. This probably results from traditional trust in dollar (hard currency) savings under communism and the unstable inflation rates in the early years of transition to the market economy. Carry traders are not only exposed to currency exchange rate risk but also have to adapt to interest rate fluctuation, inconsistent with their home country’s financial and economic situation but reflecting the national market situation for their mortgage currency. Additionally they face major risk to the value of their assets and liabilities. As Cooper (2000, p. 296) claims: “For an open economy, though, the exchange rate is also the most important price in the market for goods and services. Jumping asset prices can badly disrupt the markets on which the economic well-being of the majority of residents depend”.

**Fig. 1:**
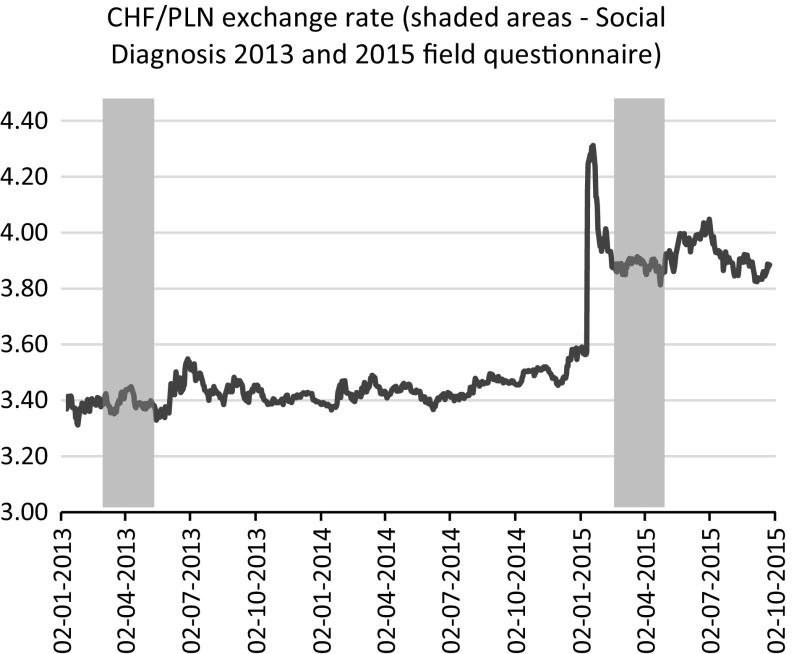
Swiss franc/Polish zloty exchange rate—Jan. 2013 to Sept. 2015

**Fig. 2:**
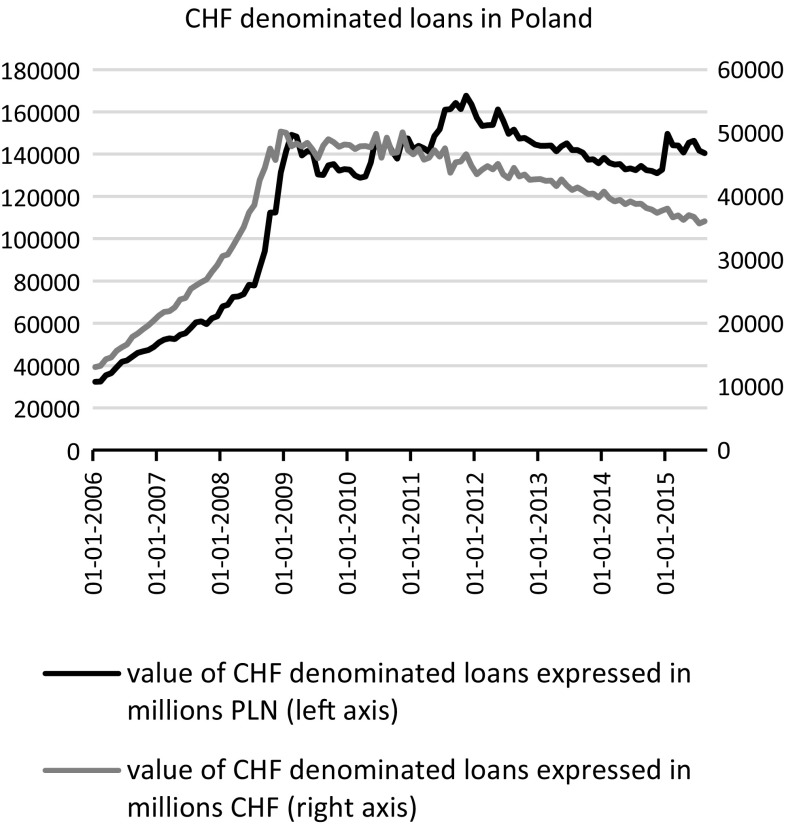
Polish zloty valuation of Swiss franc denominated loans (millions)—Jan. 2006 to Mar. 2015

In light of this, since at the beginning of 2015, the Swiss National Bank (SNB) discontinued a peg of 1.2 CHF/EUR, leading to subsequent appreciation of the Swiss currency against major currencies, including the Polish zloty (see Fig. 1), the consequences had to be dealt with by many Polish households, in terms of the considerable increase in the local currency value of these loans (Fig. 2). The question posed in this study was therefore: “When the financial risk associated with carry trade in mortgages materializes, do consumers also experience non-financial costs?” If this is true, then naturally such loans should be scrutinised and banks should at least partially account for an unexpected external effect on their customers associated with increase in the value of mortgages. However, if not, it might be that the social costs of foreign denominated mortgages are not as high as depicted. Maybe the cushion associated with the parallel interest rate reduction is sufficient and households with Swiss franc mortgage loans are not inherently worse-off.

To investigate this situation, the influence of the sudden appreciation of the Swiss franc in Jan. 2015 on the psychological well-being and physical health of Swiss franc indebted Poles was examined. To this end, two recent waves of the Polish household panel survey Social Diagnosis were used. This biennial survey investigates not only the financial situation of households, providing information about credit, its sources and intended use but also enquires about quality of life, well-being and happiness (Czapiński 2013). Data were collected in 2013 and 2015, shortly following the decision by the SNB to cease protection of the Swiss currency from appreciation (see Fig. 1). In order to investigate the impact of sudden Swiss franc appreciation, a difference-in-differences matching estimator was applied (Caliendo and Kopeinig 2008; Heckman et al. 1997, 1998; Smith and Todd 2005).

To the best of our knowledge, this paper is the first to examine how changes in the value of Swiss franc denominated mortgages after Jan. 2015 influenced the psychological and physical well-being of any group of borrowers. Since our focus is primarily on Polish mortgage holders indebted in Swiss francs, the study describes the situation of one of the groups most vulnerable to this kind of adverse financial conditions.

The paper first briefly explores the background to carry trade in Poland. Second, the data source and the measures investigated are presented. The methods applied to the analysis of the impact of Swiss franc denominated mortgages on the well-being of households are explained leading to presentation of the results. Finally, the discussion suggests further directions for research, caveats to the findings and implications to policy.

## Background

Swiss franc (CHF) denominated mortgages have become a very important product on the financial landscape in Eastern European countries. In Poland and Hungary, in 2007, their total value already exceeded 30 billion CHF (Brown et al. 2009) and grew further until the onset of the financial crisis, as many carry traders had seized the chance to improve their living standards at seemingly low expense. Of the main reasons for carry trade, the popularity of mortgages denominated in Swiss francs should most probably be attributed to the lower interest rates they offered. Since mortgages, as products, require a large contribution from monthly interest instalments (the longer the term of repayment, the higher the influence of interest rates), a 2–3 % point difference in the interest rate translates into 20–40 % savings on the monthly instalment. The supply side (banks) also willingly supported carry traders, as they provided revenue not only through the interest margin but also on exchange rate spreads (instalments are very often serviced in domestic currencies).

Significant growth in the market share of Swiss franc denominated mortgages in Poland and in the number of Polish households with Swiss franc mortgage loans was also propelled by the strengthening Polish zloty. The most rapid appreciation was observed just before the crisis. In 2007, the Polish zloty appreciated against the Swiss franc by 10 % and in the first half of 2008 by another 10 %. Although appreciation of currencies of countries on the convergence path can be substantiated by the Balassa-Samuelson effect (Balassa 1964; Samuelson 1964), if the trend exceeds the theory implications, it is likely that a change in expectations or demand would trigger a sharp trend reversal (Galati et al. 2007). This is what happened in Poland at the onset of the financial crisis. Between August 2008 and March 2009, the Polish zloty depreciated by 60 %.

Although, by 2009, lending in Swiss francs had almost stopped, the value of the Polish household debt expressed in Polish zloty further rose (Fig. 2). In consequence, the debate was initiated on the influence of foreign currency denominated mortgages on economic and social conditions of households. In fact, the two countries most affected by franc loans, either escaped (Hungary) or tried to ease the household impact (Poland) from the shock. In both countries, regulations allowing some exemption from high debt burden are either in place (Hungary) or have been discussed in parliament (Poland) (Sejm Rzeczpospolitej Polskiej VII kadencja 2015). This legislation penalizes banks for benefiting from recent depreciation of local currencies and requires that they bear most of the costs of reducing the burden of debt.

These one-sided interventions arise from the belief that availability of foreign currency denominated loans is mostly driven by the supply side-banks—and not from demand—the customers (Crespo Cuaresma et al. 2014). However, various studies show that carry traders are reasonably well prepared for adverse financial events. In Austria, foreign currency denominated mortgages are more often favoured by risk seeking, affluent, and married individuals (Beer et al. 2010). In Hungary, foreign currency borrowers are also more affluent. Additionally, they are better educated. Yet, in contrast to Austrian carry traders, this group is more risk-averse (Pellenyi and Bilek 2009). Studies of the Polish market show that those with mortgages are generally in a much better financial situation than other households (Białowolski 2014). However, when the CEE countries are analysed together, it is found that foreign currency borrowers are mainly young and do not differ from domestic currency borrowers with respect to income, education or aversion to risk (Fidrmuc et al. 2013). This contradicts the findings of Beer et al. (2010) for Austrian households.

The daily press is largely unequivocal in its assertion that impact from foreign currency denominated mortgages on psychological well-being and health is negative. Despite also being supportive to these arguments, relevant scientific theories are not indisputable. According to the economic theory of revealed preferences (Samuelson 1938), life circumstances related to changes in income (growth of income or becoming unemployed) have a persistent influence on subjective well-being, with the implication that decrease in income is associated with detriment to well-being. From this perspective, increase in debt should negatively correspond to life-time utility and well-being. In the behavioural approach to economics it is claimed that negative shocks should particularly strain utility, with losses valued at approximately twice those of gains (Benartzi and Thaler 1999). However, sociological set-point theory posits that people react to life events, although their reaction is only short term because in the long term, due to adaptation, the baseline level for well-being is regained (Easterlin 2003; Headey 2008; Lucas et al. 2004). All these arguments indicate, however, at least a short term negative effect on psychological well-being and health following an adverse shock change in the value of foreign currency denominated mortgages. Depending on which effects prevail, the shock might have long-lasting effects (revealed preferences or behavioural approach) or short-term effects only (set-point theory).

## Methods

### Data

In this study two recent waves (2013 and 2015) of the Polish household panel study ‘Social Diagnosis’ were used. This biennial study collects data on Polish living conditions and quality of life and is representative with respect to age, gender, class of place of residence and NUTS 2^2^ region (Czapiński 2013). The 2013 wave provided information about the situation of Polish mortgaged households before the sudden appreciation of the Swiss franc in Jan. 2015. The 2015 wave,^3^ instead, offered a unique window of opportunity to examine the situation very soon after it occurred.^4^ The correspondence between the Social Diagnosis 2013 and 2015 waves and changes in the CHF/PLN exchange rate is charted in Fig. 1.

The analysis addressed the situation of 1159 Polish mortgage borrowers aged 25 and over, who in 2013 and 2015, had either Polish zloty (837) or Swiss franc (322) mortgages, i.e., were exposed to the sudden appreciation of the Swiss franc. The choice of the waves to be used was determined by the availability of a question on mortgage ownership and denomination. It was available only in the 2015 wave. Consequently, it precluded examination of effects of a surge in the CHF/PLN exchange rate having occurred in 2008–2009.

### Measures

#### Outcome Variables

Three complementary outcomes were considered: (1) self-reported health (SRH), (2) medical conditions reflected by the number of somatic symptoms presenting in the month preceding the survey and (3) willingness to live. SRH is measured on a 6-point Likert scale (1-extremely dissatisfied, 2-very dissatisfied, 3-somewhat dissatisfied, 4-somewhat satisfied, 5-very satisfied, 6-extremely satisfied) and expresses psychological well-being. The incidence of somatic symptoms was measured using the Patient Health Questionnaire (see: PHQ-15 in ”Appendix”) (Kroenke et al. 2002; Yeung and Deguang 2002), assessing the presence and severity of common symptoms, among the most widely used and best-validated self-report measures for somatic symptom burden (Gierk et al. 2014). The scale ranges from 0 to 30 and mirrors physical health condition. Willingness to live (WL) is measured with a single question (At present, how strong is your willingness to live? 1-I do not want to live at all and 10-I want to live very much) and reflects a different dimension for psychological well-being. Three models, differing with respect to outcome variable were used to explore the data (Model 1 for SRH, Model 2 for PHQ-15, Model 3 for WL).

#### Treatment and Control Group

People who reported themselves as having either a Swiss franc or Polish zloty denominated mortgage in 2015 were accordingly classified into the treatment and control groups.

#### Control Variables

A rich set of control variables, already established as influential on psychological well-being, health and borrowing, were used to investigate the influence on psychological well-being and physical health of the Swiss franc borrowers—treatment group—as opposed to the Polish zloty—control group. These variables were measured before treatment—the exchange rate shock—in order to avoid endogeneity issues (Gebel and Voßemer 2014). Specifically, sociodemographic variables were controlled for, i.e., gender, age, place of residence (rural areas, small towns and cities) and type of household (childless marriage, marriage with children, one-parent family and others). These have previously been shown to correlate with both psychological well-being (Grossi et al. 2012), health (Löwe et al. 2008) and mortgage status (Burgard et al. 2012). Family support was also accommodated in this study^5^ in the belief that it might be influential in the relief of financial distress (Georgarakos et al. 2010). The analysis accounted for financial conditions such as equivalised household disposable income (after log transformation), value of debt in terms of monthly income, the monthly instalment as a percentage of monthly household income and labour market status (employed, unemployed and economically not active).^6^ Finally, differences in 2013 health conditions were also captured to ensure balancing properties in this respect. The question about activity limitations owing to health problems was therefore introduced. It should be inferred that, following the reasoning of Lechner (2010) and further applied by Gebel and Voßemer (2014), conditioning was not on the pre-treatment outcome itself, as this could introduce a correlation with the treatment and, thus, violate the common trend assumption.

### Statistical Methods

Using longitudinal data from the Polish Social Diagnosis 2013–2015 surveys, the strengths of two approaches for evaluation of causal inference were combined. These were: (1) propensity score matching (PSM), which attempts to eliminate selection bias by conditioning on confounding variables and past health and (2) the difference-in-differences (DID) estimator (Caliendo and Kopeinig 2008; Heckman et al. 1997; Lechner 2010; Smith and Todd 2005), which removes unobserved fixed effects via intra-individual comparisons over time by comparing trends in a treatment and control group. The strategy described by Austin (2011a, b) was followed and the psmatch2 function (Leuven and Sianesi 2003) in Stata 14 was used for analysis.

As domestic currency borrowers may substantially differ from foreign currency borrowers, being more prone to preselection by pre-existing financial or health issues, simple control for confounders might not be adequate to distinguish a causal relationship between the Swiss franc exchange rate surge in Jan. 2015 and the outcomes. This meant that in order to circumvent this problem a technique was needed to match Swiss franc borrowers with those with Polish zloty mortgages. This would be conditioned on the observed initial 2013 characteristics, which had affected both health and well-being outcomes, beyond debt status, debt value and monthly repayments.

Matching was based on similarity of propensity scores, calculated using a probit regression model and the control variables, previously mentioned and detailed in Table 1. All control variables were retained in the prediction equation even if proved non-significant for prediction of outcomes (Quesnel-Vallée et al. 2010; Rubin and Thomas 1996), though most proved significant. Statistical twins, based on propensity scores, were found using the 5-nearest neighbours matching algorithm with replacements. This strategy allowed the treatment and control groups to be balanced with respect to the observed characteristics. This improved the plausibility of the common trend assumption (Gebel and Voßemer 2014). Covariate balance between the treatment and control groups was observed using both standardized differences and percentage bias reduction (Austin 2011a; Gebel and Voßemer 2014).

**Table 1:** Covariate balancing: mean and proportion difference before and after matching

Control variable	Treated	Untreated	Std diff	% red bias
*Gender*				
Female (ref: male)				
Before	0.56	0.55	0.6	
After	0.55	0.59	−7.0	−1049.1
Age				
Before	40.04	43.40	−31.7	
After	40.15	39.85	2.8	91.2
*Place of residence (ref: cities)*				
Towns				
Before	0.35	0.39	−8.6	
After	0.36	0.37	−2.3	73.9
Rural areas				
Before	0.27	0.35	−17.7	
After	0.28	0.25	6.8	61.3
*Type of household (ref: childless marriage)*				
Marriage with children				
Before	0.76	0.66	21.3	
After	0.77	0.78	−3.6	82.9
One-parent family				
Before	0.02	0.06	−19.7	
After	0.02	0.02	1.7	91.5
Others				
Before	0.06	0.16	−29.7	
After	0.07	0.08	−3.5	88.1
*Feeling loved and trusted (ref: yes)*				
No				
Before	0.05	0.04	6.3	
After	0.05	0.05	1.6	75.5
Ln (equivalised household disposable income)				
Before	7.74	7.45	49.7	
After	7.71	7.74	−4.2	91.5
*Activity limitations due to health problems (ref: very often)*				
Sometimes				
Before	0.44	0.44	−0.2	
After	0.44	0.45	−1.3	−530.8
Never				
Before	0.49	0.46	5.8	
After	0.49	0.49	0.4	92.5
*Household total debt related to monthly incomes (ref: debt below 12 monthly incomes)*				
Debt ranging from 12 to 36 monthly incomes				
Before	0.12	0.15	−9.1	
After	0.13	0.11	4.1	54.6
Debt exceeding 36 monthly incomes				
Before	0.71	0.38	69.8	
After	0.70	0.70	1.6	97.7
*Monthly repayments related to monthly incomes (ref: up to 20* *%)*				
20–40 %				
Before	0.34	0.29	10.4	
After	0.34	0.39	−10.6	−1.8
Above 40 %				
Before	0.12	0.09	10.6	
After	0.13	0.10	7.8	26.7
*Labour market status (ref: employed)*				
Unemployed				
Before	0.03	0.03	−4.3	
After	0.03	0.03	0.6	85.1
Employed				
Before	0.12	0.17	−14.0	
After	0.12	0.14	−4.4	68.6

*std diff* standardised difference;  *% red bias* bias reduction expressed in percentages

Changes in the psychological well-being and physical health outcomes between CHF mortgage holders (treated) and their matched PLN counterparts (control) were then used to estimate the average treatment effect on the treated (ATT). ATT corresponds to the impact on psychological well-being and health of households with Swiss franc denominated mortgages from the Swiss National Bank decision of Jan. 2015 to abandon its 3-year cap of 1.20 Swiss francs per euro. To assess the significance of the ATT more reliably, bootstrapping was used with 1000 draws to estimate the standard error for ATT.

Following the recommendation of Austin (2011b), sensitivity analysis was conducted. We tested differing numbers of neighbours in the matching algorithm (1–5) and matching with the calliper algorithm. Analysis was additionally performed by subgroup, according to level of education (higher education and below) and income bracket (2015 monthly disposable household income [PLN]—below 3000 and over 3000). Results (available upon request), were highly robust to these modifications (always non-significant in terms of ATT).

## Results

Three probit regression models using control variables as in Table 1 were built (see: Table 3 in Appendix). No control variables were rejected from the models even if identified as non-significant. The pseudo R^2^ values were calculated, 0.141 for the SRH model, 0.142 for WL and 0.146 for PHQ-15. Due to missing dependent or control variables, the final sample for analysis comprised 714 observations for the SRH, 686 for the PHQ-15 and 715 for the WL probit regression models.

Matching results are presented in Table 1. For continuous control variables, the average is presented and for categorical control variables—proportion of treated and untreated, before and after matching. No more than 4 observations per model were outside the common support range and therefore excluded from analysis.

Examination of the “before” estimates shows that Swiss franc and Polish zloty borrowers differed noticeably in terms of income—most importantly Poles from CHF indebted households varied with respect to the initial value of the debt and debt servicing expenses. They were over 3 years younger than those with PLN loans who were from households with 25 % lower *per capita* incomes. Of CHF borrowers, the proportion with debt exceeding their triennial household income was over 71 %, while for PLN borrowers—only 38 %. Additionally, individuals whose households spent over 40 % of their income on debt servicing accounted for 12.3 % in the treatment and 9.0 % in the control groups. Differences between control and treatment groups before matching were also major when place of residence was taken into account. Of PLN borrowers, more individuals lived in rural areas compared to those indebted in CHF (PLN—35.5 % vs. CHF—27.3 %). Those having PLN denominated mortgage were also more likely to live in small towns than those indebted in CHF (39.5 vs. 35.3 %). So, it appears that respondents with CHF loans lived in cities more frequently than respondents with PLN loans.

Additionally, type of household distinguished both groups. Before matching, of CHF borrowers, the proportion of married couples both with and without children was higher than for PLN borrowers. The proportion of single parent families was higher among PLN borrowers. Finally, individuals from CHF indebted households were less likely to be unemployed (2.7 vs. 3.4 %) and also much less frequently outside the labour force (11.8 vs. 16.6 %). Little difference, even before matching, was observed with respect to family support and activity limitations due to health problems.

The treatment and control groups did not considerably differ after matching. As the standardized differences for matched individuals indicate, almost all differences were well below 10 %.^7^ This implies that, due to matching, all predictors, which prior to matching were above the 10 % threshold, showed significant improvement in terms of bias. The only predictors with increased bias were those which initially showed very low bias but which still remained acceptable even after matching.

Very good matching was also confirmed by the likelihood ratio test. For the unmatched sample the LR Chi square statistic showed 117.05 yielding *p* = 0.000, while for the matched sample the outcome was LR = 3.58 with *p* = 1.000. From this it should be implied that the hypothesis of similarity between unmatched samples was rejected, while PSM allowed almost all differences between the treatment and the control groups were accounted for.

**Fig. 3:**
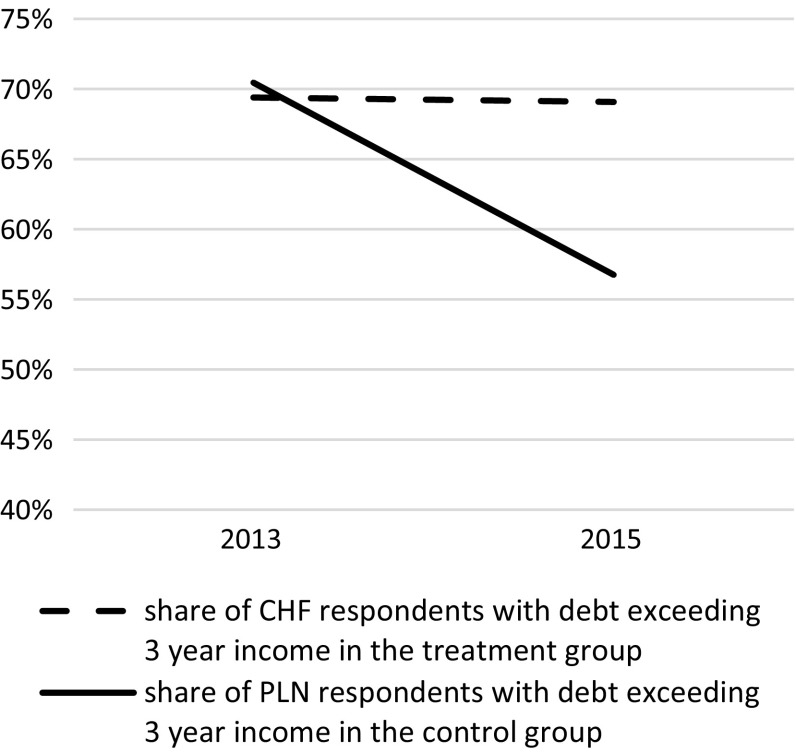
Poles with debt exceeding 3 year income—indebted in Polish zloty (matched treatment group) and Swiss francs (control group)

**Fig. 4:**
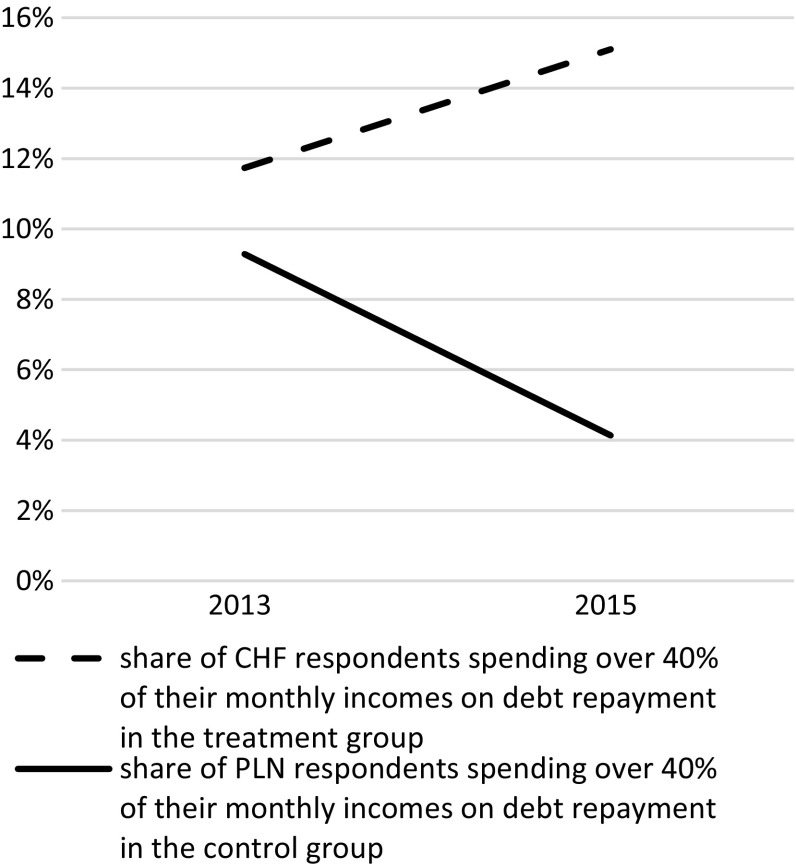
Poles spending over 40 % of monthly income on mortgage debt–debt in Polish zloty (matched treatment group) and Swiss francs (control group)

To demonstrate that the impact of the Jan. 2015 Swiss National Bank decision on Poles proved to be extremely significant from the financial perspective, evolution of the value of debt and instalment repayment in relation to equivalised disposable household income was examined in both treatment and matched control groups (Figs. 3, 4).

In 2013, individuals in households with debts exceeding their triennial income was equalised at a proportion of ca. 70 % for both the treatment and the matched control group. However, for the same groups, 2 years later and after the Swiss franc appreciation in Jan. 2015, the proportion of respondents with CHF denominated mortgages and in debt exceeding their triennial incomes remained the same, while in the matched control group (matched holders of Polish zlotys mortgages) it dropped to 57 %. Even more divergent trends, despite affecting far fewer individuals, were observed with instalments. Between 2013 and 2015, CHF mortgage holders spending over 40 % of their monthly incomes on instalments increased from 12 to 15 %, while those indebted in PLN and contributing over 40 % of their incomes reduced from 9 % to a mere 4 % (in the matched control group).

This clearly suggests that the overall financial burden associated with mortgage debt increased for those indebted in Swiss francs, while it significantly eased for those repaying their PLN debts. Did the burden of debt in Swiss francs also impact psychological or physical aspects of individual well-being, i.e., health and willingness to live? In order to find out, a difference-in-difference model was estimated and the treatment and control groups assessed with respect to change in self-reported and physical health, in addition to willingness to live. The results are presented in Table 2.

**Table 2:** ATT estimates

	ATT	SE	Z	*p* value	95 % CI
Model 1—SRH	−0.178	0.132	−1.34	0.181	(−0.438; 0.083)
Model 2—PHQ-15	0.078	0.486	0.16	0.873	(−0.875; 1.030)
Model 3—WL	−0.104	0.176	−0.59	0.556	(−0.448; 0.241)

SRH is measured on a 6-point Likert scale (1-extremely dissatisfied, 2-very dissatisfied, 3-somewhat dissatisfied, 4-somewhat satisfied, 5-very satisfied, 6-extremely satisfied); PHQ-15 ranges from 0 to 30; WL is measured on 1–10 scale

Coefficients reported for all difference-in-difference effects, although pointing to relative worsening of the situation for Swiss franc mortgage owners, were very close to 0 and non-significant. This implies that although Poles with debts in Swiss francs are presently more disadvantaged financially, as compared to their situation in 2013, the same cannot be confirmed with respect to their psychological well-being and health.

## Discussion and Conclusions

This study sought to reveal the causal effects of CHF/PLN exchange rate change on individual well-being and health. To this end, the Polish representative household panel survey Social Diagnosis was used (waves 2013 and 2015) and difference-in-difference matching estimator was applied (Caliendo and Kopeinig 2008; Heckman et al. 1997; Smith and Todd 2005). This avoided selection problems for observable and unobserved fixed characteristics. Results indicated that, although Poles indebted in Swiss francs experienced considerable detriment to their financial situation, change to the CHF/PLN exchange rate had no significant influence on their well-being, health and willingness to live. It showed that carry traders may require a long period for negative psychological consequences of a negative exchange rate shock to become manifest, if such ill-effects indeed exist. In the short run, adverse financial circumstances, even when considerable variation in debt value was experienced, did not significantly affect health or willingness to live. In the longer run, when individuals have more time to truly appreciate their situation, these conclusions may change.

This study deepens understanding about the well-being and health of mortgage borrowers. First, based on our study we can argue that although housing instability, mortgage indebtedness and mortgage foreclosure can be to the detriment of subjective well-being (Nettleton and Burrows 1998) and self-rated health (Cannuscio et al. 2012; Lau and Leung 2014) as well as increased propensity to anxiety attacks and depression (Burgard et al. 2012), a short-term substantial change in the burden of debt had no significant influence on psychological and physical health. Second, although debts are a source of significant risk and distress (Brown et al. 2005; Sweet et al. 2013), Polish carry traders seem to be psychologically resilient to currency exchange rate risk, at least in the short term. Alternative explanations are also possible. Most Polish carry traders had already experienced a considerable negative shock associated with an increase of the CHF/PLN exchange rate. Their financial standing had already deteriorated in 2008–2009 during the first wave of Swiss franc appreciation. Consequently, in 2015 they might have already been inured to this type of shock. It may also be that they were sufficiently financially literate to understand the short term benefits of interest rate fluctuations and hence did not become anxious about exchange rate fluctuations or lacked financial literacy to such an extent that they were entirely unaware of the risk of foreclosure. Finally, it might have been that lower interest rates in the short run balanced the burden of much larger debt. Further research is needed in order to understand the truth.

The study reveals that after a loan is granted, even considerable short-term change in value might not affect borrowers’ psychological well-being or physical health. These conclusions cast doubt on relevance of the economic theories: either of revealed preferences; of behavioural economists or being the sociological set-point theory, which predict at least short term negative effects on the well-being of mortgage borrowers.

Some caveats apply to the findings and conclusions presented. Firstly, the conclusions relate only to Poland, where, it should not be underestimated, mortgages are distributed among the most affluent households, with high incomes and good debt service histories (Białowolski 2014). In these cases, even a considerable change in debt burden would not immediately reduce a household standing to a level that would threaten health; especially if the financial burden (associated with increase in debt) was with an associated significant parallel reduction in the interest rates by the Swiss National Bank on the same day, as occurred in the case to point. A second limitation relates to the fact that in this analysis, information about mortgage ownership in 2013 was extrapolated from the mortgage data from 2015, which was directly available from the survey. For the same reason the study did not assess the influence of the surge in the CHF/PLN exchange rate in the years 2008–2009. This may have influenced the results here as the possibility cannot be excluded that Swiss franc indebted Poles having experienced a CHF/PLN exchange rate surge in 2008–2009, had adapted to the situation (e.g., austerity, search for a second job, borrowing from relatives or friends) and knew what to expect in at least the short run. However, it may also mean that they had already used up all alternative sources of income in the past and in the longer perspective might expect negative consequences. Thirdly, there is long-standing debate on the understanding and usefulness of subjective compared with objective measures (Białowolski and Węziak-Białowolska 2014), especially the use of self-rated health (Fayers and Sprangers 2002; Jylhä 2009; Węziak-Białowolska 2014). Although these measures are believed to provide useful information, concerns about understanding of concepts and the assessment process threaten the validity of results. Fourthly, as it is plausible that carry traders are less risk averse, their exposure to negative impact of debt fluctuations might be limited. However, due to lack of appropriate data, it was not, in this study, possible to include risk aversion in the matching model. Finally, as previously mentioned, no short-term effect from sudden appreciation of the Swiss franc on psychological well-being and health was detected but this, however, does not preclude longer term impact.

Our findings also suggest policy recommendations. Since, in the next 20–30 years, many Polish households with foreign currency denominated mortgages will remain exposed to the risk of exchange rate instability, improvement in financial literacy is needed. It is especially important to educate people about the influence of exchange and interest rates on the value of debt and repayment instalments. Additionally, as carry traders have already suffered severe negative financial effects, their well-being and health should be further monitored, since longer term effects might still be revealed.

## Appendix: Patient Health Questionnaire (PHQ-15)

In the past month: 0—I did not suffer; 1—I suffered less than 15 days; 2—I suffered at least for one half of the month

**Table:**

1. Strong headaches	9. Accelerated heartbeat (palpitation)
2. Stomach pains or flatulence	10. Shivers or convulsions
3. Pain or tension in the neck or arm muscles	11. Pressure on the bladder and more frequent urinating
4. Chest or heart pains	12. A feeling tiredness not associated with work
5. Dry mouth or throat	13. Constipation
6. Attacks of excessive sweating	14. Nosebleeds
7. Shortness of breath	15. Sudden changes of blood pressure
8. Pain in your arms, legs, or joints (knees, hips, etc.)	

See Table 3.

**Table 3:** Estimates of the probit model for treatment

Model	SRH	PHQ-15	WL
Control variable			
Female (ref: male)	0.047	0.047	0.055
Ln(equivalised household disposable income)	0.448***	0.473***	0.454***
Age	−0.009	−0.011*	−0.008
Place of residence (ref: cities)			
Towns	−0.315**	−0.260*	−0.310**
Rural areas	−0.209	−0.187	−0.207
Type of household (ref: childless marriage)			
Marriage with children	0.01	−0.054	0.012
One-parent family	−0.645*	−0.662*	−0.643*
Others	−0.538**	−0.551**	−0.531**
Feeling loved and trusted (ref: yes)			
No	0.385	0.39	0.444*
Activity limitations due to health problems (ref: very often)			
Sometimes	0.215	0.217	0.224
Never	0.034	0.027	0.056
Household total debt related to monthly incomes (ref: debt below 12 monthly incomes)			
Debt ranging from 12 to 36 monthly incomes	0.354**	0.345*	0.355**
Debt exceeding 36 monthly incomes	0.885***	0.906***	0.892***
Monthly repayments related to monthly incomes (ref: up to 20 %)			
20–40 %	−0.124	−0.111	−0.128
Above 40 %	0.203	0.11	0.201
Labour market status (ref: employed)			
Unemployed	−0.01	0.051	−0.006
Employed	0.178	0.19	0.155
Constant	−4.056***	−4.125***	−4.171***
Pseudo R^2^	0.141	0.146	0.142
Number of observations	710	686	715

*** Coefficients significant at 0.01 level; ** coefficient significant at 0.05 level; * coefficient significant at 0.1 level

## Footnotes

1 Those who willingly take out loans in foreign currency in order to benefit from (1) lower interest rates and (2) lower volatility are called carry traders.

2 The NUTS classification (Nomenclature of territorial units for statistics) is a hierarchical system for dividing EU economic territory to collect, develop and harmonise the European regional statistics. Several layers are distinguished. NUTS 0 correspond to countries, NUTS 1 are major socio-economic regions, NUTS 2 are basic regions for the application of regional policies. NUTS 3 are small regions for specific diagnoses. In Poland NUTS 2 regions are called voivodeships.

3 Collection of the data was conducted between March and June 2015.

4 It is also worth underlining that in 2015 the currency exchange rate shock, subject to investigation here, was much more isolated than the shocks in 2008/2009. At the onset of the financial crisis, a variety of factors played a role (international turbulence, decreased GDP growth rate, financial sector instability, etc.). All these factors might have had differentiated effects on mortgage holders with different mortgage denominations. On 15th January 2015 almost nothing else but the CHF/PLN exchange rate changed, which provides additional argument for our analysis.

5 Family support was addressed with a question “do you feel loved and trusted?” (yes/no).

6 However, due to ambiguous impact of risk-aversion on possession of foreign currency denominated (see Sect. 2), we do not condition on any proxy of this measure. Moreover, in the Social Diagnosis surveys 2013 and 2015 there were no risk-aversion related questions available.

7 The only exception was the proportion of individuals from households with monthly instalments ranging from 20 to 40 % of their incomes. It was just above that cut-off point.
